# Usefulness and acceptability of a standardised orientation and mobility training for partially-sighted older adults using an identification cane

**DOI:** 10.1186/1472-6963-12-141

**Published:** 2012-06-08

**Authors:** Judith Ballemans, GA Rixt Zijlstra, Ger HMB van Rens, Jan SAG Schouten, Gertrudis IJM Kempen

**Affiliations:** 1 Department of Health Services, Research, and CAPHRI School for Public Health and Primary Care, Faculty of Health Medicine and Life Sciences, Maastricht University, Maastricht, The Netherlands; 2 Department of Ophthalmology and the Institute for Research in, Extramural Medicine (EMGO), Amsterdam, The Netherlands and Department of Ophthalmology Elkerliek Hospital Helmond, VU University Medical Center, Amsterdam, Netherlands; 3 Department of Ophthalmology, University Hospital Maastricht, Maastricht, The Netherlands

**Keywords:** Low vision, Aged, Mobility limitation, Canes, Process assessment

## Abstract

**Background:**

Orientation and mobility (O&M) training in using an identification (ID) cane is provided to partially-sighted older adults to facilitate independent functioning and participation in the community. Recently, a protocolised standardised O&M-training in the use of the ID cane was developed in The Netherlands. The purpose of this study is to assess the usefulness and acceptability of both the standardised training and the regular training for participants and O&M-trainers in a randomised controlled trial (NCT00946062).

**Methods:**

The standardised O&M-training consists of two structured face-to-face sessions and one telephone follow-up, in which, in addition to the regular training, self-management and behavioural change techniques are applied. Questionnaires and interviews were used to collect data on the training’s usefulness, e.g. the population reached, self-reported benefits or achievements, and acceptability, e.g. the performance of the intervention according to protocol and participants’ exposure to and engagement in the training.

**Results:**

Data was collected from 29 O&M-trainers and 68 participants. Regarding the self-reported benefits, outcomes were comparable for the standardised training and the regular training according the trainers and participants e.g., about 85% of the participants in both groups experienced benefits of the cane and about 70% gained confidence in their capabilities. Participants were actively involved in the standardised training. Nearly 40% of the participants in the standardised training group was not exposed to the training according to protocol regarding the number of sessions scheduled and several intervention elements, such as action planning and contracting.

**Conclusions:**

The standardised and regular O&M-training showed to be useful and mostly acceptable for the partially-sighted older adults and trainers. Yet, a concern is the deviation from the protocol of the standardised O&M-training by the O&M-trainers regarding distinguishing elements such as action planning. Overall, participants appreciated both trainings and reported benefit.

## Background

Low vision associated mobility restriction is a common problem among older adults [1,2] and is likely to result in loss of independence, increased social isolation, decreased quality of life and high levels of depression [3-6]. Orientation and mobility (O&M) training, including the use of assistive devices such as the identification (ID) cane, also called symbol cane, may help partially-sighted older adults to maintain or regain independent functioning, and to improve participation and quality of life [7-9]. An ID cane is frequently used to indicate one’s low vision to others in situations such as street crossings and crowded places [10]. This cane is white with red straps and is approximately 95 centimeters in length. Its prime function is signalling, and in contrast to the long cane (approximately 100 to 150 centimeters in length) it is less suitable for detection of low objects. The O&M-training in the use of the ID cane is currently worldwide largely practice-based. However, in general the training is unstructured and heterogeneous regarding content and format [11] and insight into the effectiveness and usefulness of such training is lacking [8,12,13].

For these reasons, a standardised O&M-training in the use of the ID cane for partially-sighted older adults was developed [11,12,14,15]. The standardised O&M-training aims to facilitate safe and independent participation in the community, optimal use of one’s abilities and to facilitate uptake of old or new activities [14]. The training is theory-driven and pre-structured in a protocol to facilitate equal application of the training by the mobility trainers. Yet, the training is also tailor-made to each partially-sighted individual via the applied behavioural change strategies such as setting personal goals, individual problem-solving and finding personal, realistic solutions. This enables meeting the specific needs of a participant regarding orientation and mobility skills during the training. However, it is unknown how mobility trainers and partially-sighted participants regard the new O&M-training in terms of usefulness and acceptability compared to the regular O&M-training. To study this process evaluations are performed to recognize strong and weak aspects of an intervention, to explain observed (lack of) intervention effects, and to further improve an intervention prior to implementation, i.e. if an intervention turns out to be effective [16,17].

This study presents the outcomes of a process evaluation, which was carried out to provide insight into the usefulness (feasibility) and acceptability of the standardised and regular O&M-training in the use of the ID cane as perceived by mobility trainers and partially-sighted older adults. The process outcomes include: 1) the population reached (usefulness), 2) the self-reported benefit or achievement (usefulness), 3) experienced barriers and potential solutions (usefulness), 4) the extent to which the intervention was performed according to protocol (acceptability), 5) the participants’ exposure to and engagement in the training (acceptability), and 6) the opinion about the training (acceptability).

## Methods

### Study design

A randomised controlled trial (NCT00946062) was conducted in collaboration with the two main (not-for-profit) organizations for low vision care in The Netherlands: ‘Bartiméus’ and ‘Royal Dutch Visio’ (the latter was previously known as the two organizations ‘Sensis’ and ‘Visio’) [12]. In The Netherlands older adults with low vision are referred to these centers by a low vision specialist, ophthalmologist, general practitioner, friend or family member, or seek the professional advice available in these centers themselves. The costs of the O&M-training are reimbursed, but the costs of the ID cane (approximately € 25) are not. In 18 local centers, scattered over the country, O&M-training in the use of the ID cane is provided by certified O&M-trainers to partially-sighted older adults.

In the trial the centers were randomly allocated to the intervention or control status, standardised and regular O&M-training, respectively, and stratified by organization [12]. O&M-trainers, researchers and participants were not masked for intervention status, owing to the nature of the trial and the general information on the trial provided to participants before enrolment into the study. However, participants received no information regarding their intervention status after randomisation. Telephone interviews were performed by trained interviewers who were masked for group allocation and who collected data from the participants (i.e. partially-sighted older adults).

The Medical Ethical Committee of the Maastricht University/Academic Hospital Maastricht granted approval for conducting this study and the research adhered to the tenets of the Declaration of Helsinki.

### Study population

#### participants

The screening of potential participants started in November 2007 and occurred in a stepped procedure [12]. *Firstly*, during the low vision center’s standard exploratory interview a staff member of a local centre roughly screened a potential participant using a six-item pre-structured registration form. The items included refer to whether the participant: 1) experienced low vision, 2) was 55 years of age or older, 3) lived independently or in a home for older people, 4) experienced difficulty to avoid large obstacles due to vision loss, 5) had additional impairments that cause full inability to leave the home, and 6) consented to an additional screening interview by telephone for potential participation in the trial. If items 1, 2, 3 and 6 were confirmed with ‘yes’ and items 4 and 5 were confirmed with ‘no’, contact information was recorded and the registration form was sent to the research team. *Secondly*, a pre-structured 10-minute interview by telephone was conducted by the research team to assess the participant’s eligibility for participation in the trial. The additional inclusion criteria were: 1) able to go outside for a short walk or doing groceries, and 2) experiencing difficulties with safely crossing a street, and/or experiencing difficulties with recognizing acquaintances outdoors, and/or willing to become recognizable as partially-sighted by means of the ID cane. *Lastly*, O&M-trainers of the selected local centers decided during a personal interview with the participant whether O&M-training in the use of an ID cane was the proper care for the participant [12].

Participants were excluded if at least one of the following criteria was met: a) cognitive impairment (a score of less than 4 on the Abbreviated Mental Test 4 (AMT4) during an interview by telephone) [18,19], b) unable to complete an interview by telephone due to language or hearing problems, c) unable to participate in or finish the O&M-training due to confinement to bed or potential nursing home admission, d) permanent use of a walking aid that is incompatible with the use of an ID cane (such as a support cane), e) having recently received an O&M-training in the use of an ID cane and permanent use of this cane outdoors, and f) not receiving an O&M-training in the use of an ID cane as treatment for their mobility problem [12].

#### O&M-trainers

O&M-trainers were eligible if they provided O&M-training in the use of the ID cane to partially-sighted older adults in one of the low vision organizations. Trainers who facilitated the standardised O&M-training received a two-hour instruction by author GZ. Twelve O&M-trainers facilitated the standardised training in the intervention group and 17 O&M-trainers facilitated the regular training in the control group. To prevent exposure of the trainers in the control group to the standardised training of the intervention group, randomisation stratified by organization was performed at the level of the local centers. More details about study design and eligibility of participants and O&M-trainers are reported elsewhere [12]. To encourage and maintain active participation of the local centers, the staff received regular updates (newsletters, monitoring telephone contacts, and weekly reviews by email) from the research team with information about the number of participants and their progress in the trial.

### Orientation and mobility training

Participants allocated to the control group received care as usual, i.e. the regular O&M-training in the use of the ID cane, whereas participants in the intervention group received the standardised O&M-training in the use of the ID cane. No restrictions were held with respect to receiving other care.

#### Regular training (usual care; control group)

The regular O&M-training in the use of the ID cane lacks a pre-structured protocol, and is thus practice-based. A previous field study with O&M-trainers in The Netherlands (n = 18) reported on this training [12,14]. On the basis of these results, it was expected that, in general, this training consists of one or two face-to-face sessions in the participants’ home environment and that the content, format and duration of the sessions varies substantially. However, three elements were expected, i.e. 1) exploration of participant’s needs, 2) providing information (e.g., on walking aids, canes and techniques related to orientation and safe behaviour), and 3) training techniques related to orientation and safe behaviour outdoors while the participants applied the ID cane. Data retrieved from the current study will provide insight into the O&M-training as usual as conducted by the trainers in addition to the standardised training.

#### Standardised training (intervention group)

The standardised O&M-training program in the use of the ID cane was developed based on a systematic review of the literature and a previous field study [11,14]. This training includes a pre-structured protocol of two face-to-face sessions, 90 and 85 minutes, respectively, and one 25-minute session by telephone. During session 1 at week 1 the participant’s needs are explored and prioritized and the trainer provides information on the ID cane (e.g., techniques related to holding the cane, orientation and safe behaviour). Subsequently, the participant formulates an action plan regarding one of the participant’s needs (e.g., what measures need to be taken to safely walk to the post office). The action plan is immediately performed outside. The trainer monitors these activities, offers assistance if required, and provides feedback. At the end of this session, the participant and trainer evaluate the action plan, make agreements regarding homework (i.e. contracting on performing and formulating new action plans) and conclude the session. During session 2 at week 3, session 1 and the homework assignments are evaluated. Next, participant and trainer perform the action plan of session 1, and formulate and perform a new action plan (e.g., a different, more challenging walking route). Again, these action plans are evaluated and agreements are made at the end of the session. During session 3 at week 5, which is held by telephone, all action plans are evaluated, a general evaluation of the training is conducted and, if necessary, a new appointment for a new session is made.

Compared with the regular training, several self-management and behavioural change techniques from theoretical frameworks, e.g. social-cognitive theory, are incorporated in the protocolised, standardised training. The added training components aim to stimulate active involvement of the client. For example, the client identifies and acknowledges difficulties regarding certain activities, recognizes different levels of difficulty and learns to set graded tasks, recognizes personal negative thoughts related to activities (including psychosocial factors) and learns how to reframe these into positive thoughts (cognitive restructuring) by searching for personal, realistic solutions to perform an activity safely (problem solving and action planning). The role of the trainer during the training includes providing information on consequences (costs and benefits of ID cane use), prompting the client to use the ID cane, assistance in action planning, setting graded tasks, identifying and overcoming barriers for cane use, providing instruction, general encouragement and specific feedback, modelling the use of the ID cane, creating positive experiences while practicing use of the cane, and discussing, evaluating and contracting regarding cane use in daily life. Several supportive materials for the trainers are provided, such as a detailed written protocol of the training (including information regarding assessment traffic speed, factors influencing sight, position of the cane before and during street crossings, and functional vision) and a checklist to mark whether the content of the training is executed. For the client two worksheets (prioritizing activities and planning activities) and a reference sheet with background information regarding the cane e.g. on when to use the cane and one’s rights and duties in traffic situations (also for significant others of the client) are included. Details of the development and contents of the standardised O&M-training are reported elsewhere [14].

### Data collection

The six main outcomes to determine the usefulness and acceptability of the O&M-training are (Table 1): 1) the population reached (usefulness), 2) the self-reported benefit or achievement (usefulness), 3) experienced barriers and potential solutions the (usefulness), 4) the extent to which the intervention was performed according to protocol (acceptability), 5) participants’ exposure to and engagement in the training (acceptability), and 6) the opinion about the training (acceptability). Between March 2008 and April 2010 these data were collected from participants and/or trainers of the intervention group and control group by means of: 1) *registration forms and interviews* to screen for eligibility, 2) semi-structured *process questionnaires* completed by the O&M-trainer for each participant after completion of the training or after a maximum of three training sessions, and 3) pre-structured 25-minute *process interviews* by telephone with the participants at 8 weeks after the start of the training. The interviews by telephone were performed by trained interviewers who were masked for group allocation. Primary diagnosis and mean functional acuity score were derived from the participants’ medical file. Several actions, e.g., requests via email and telephone, were undertaken if the questionnaires of the trainers were not returned or had incomplete data.

**Table 1:** Outcomes of the process evaluation

**Outcome measures**	**Operationalisation**	**Measurement**		
		SCR	Qt	TIp
*Usefulness*				
Population reached	General characteristics of the participants and trainers	+		
	Target population and proportion of the intended target population	+	+	
	Number of participants that refused, dropped out or completed training and reasons for withdrawal		+	
Self-reported benefit or achievement	Benefit regarding the training according to trainers		+	
	Benefit regarding the training according to participants			+
	Use of identification cane in daily life		+	+
	Achievement regarding to training goals		+	
Experienced barriers and potential solutions	Deviations of each session element*		+	
	Main goal, strong and weak aspects of the training		+	+
	Hampering and encouraging factors of the standardised training*		+	
	Matters for improvement – materials and standardised training*		+	
*Acceptability*				
Extent to which intervention was performed according to protocol	Format, preparation time and duration of the session		+	
	Per session element: performance, duration and participation by participant*		+	
	Extent to which participant achieved training goals*		+	
Participants’ exposure to and engagement in the training	Total number of sessions		+	
	Use of materials*		+	
	Opinion of trainer/participants regarding participant’s engagement		+	+
	Extent to which participants complied with contracts*		+	
	Quality of action plans formulated by participant*		+	
Opinion about the training	Overall opinion about the training by trainer and participant		+	+
	Opinion regarding number, duration and progress of the sessions by trainer and participant		+	+
	Opinion regarding comprehensibility of the training			+
	Opinion regarding number of extra sessions needed and whether the participant’s need for mobility support was met		+	+
	Burden experienced by participant		+	
	Recommendation of the training to others		+	
	Overall opinion about the trainer		+	+

SCR = *registration form and interview* to screen for eligibility; Qt = semi-structured *process questionnaire* completed by the O&M-trainer for each participant after completion of the training or after a maximum of three training sessions; TIp = pre-structured 25-minute *process interview* by telephone with the participants at 8 weeks after the start of the training.

### Data analysis

Qualitative data of the process interviews by telephone and questionnaires, i.e. answers to open questions, were classified into categories until themes and patterns in the answers emerged. Hence, if participants used different wording in their answers to describe that the trainer provided adequate information on the use of the ID cane, such a category was created and the number of matching responses was counted. Next, all quantitative data were analyzed by means of descriptive statistics, Student *t-*tests and chi-square tests (SPSS 15.0). Missing variables of partly completed questionnaires were defined as missing values.

## Results

### Usefulness

#### Population reached

##### Characteristics of participants and trainers

Table 2 shows the general characteristics of the participants and trainers. Participants’ mean age, gender, level of education, living arrangement, primary diagnosis and mean functional acuity score did not significantly differ between both groups (*p* > 0.05). Macular degeneration was somewhat more common among participants in the control group (70% vs. 55% in the intervention group). Trainers’ mean age, gender, educational background, years of working experience, and hours conducting O&M-training per week did not significantly differ between both groups (*p* > 0.05). The vast majority (75 - 88%) of the trainers were specialized occupational therapists.

**Table 2:** General characteristics of the participants and trainers per group

**General characteristics**	**Standardised training group**		**Regular training group**		***p-value***
*Participants*	(n = 31)		(n = 37)		
mean age (SD)	76.9	(8.9)	75.3	(8.6)	0.44
number female (%)	19	(61)	22	(60)	0.88
level of education (%)					0.15
low	12	(39)	16	(43)	
medium	10	(29)	17	(46)	
high	9	(32)	4	(11)	
number living alone (%)	19	(61)	17	(46)	0.16
primary diagnosis (%)					0.26
macular degeneration	17	(55)	26	(70)	
glaucoma	5	(16)	2	(5)	
other	10	(32)	7	(19)	
mean functional acuity score (range)					
right	0.15	(0.00-0.70)	0.19	(0.03-0.80)	0.32
left	0.14	(0.00-0.50)	0.19	(0.00-1.00)	0.19
binocular	0.21	(0.03-0.60)	0.22	(0.03-1.00)	0.74
*Trainers*	(n = 12)		(n = 17)		
mean age (SD)	39.0	(12.1)	33.9	(8.9)	0.20
number female (%)	11	(91)	15	(88)	0.77
occupational therapists (%)	9	(75)	15	(88)	0.37
mean years of experience (range)	8.3	(0–22)	5.7	(0–27)	0.31
mean hours O&M-training per week (range)	11.5	(2–32)	9.8	(1–40)	0.77

##### Response

**Figure 1:**
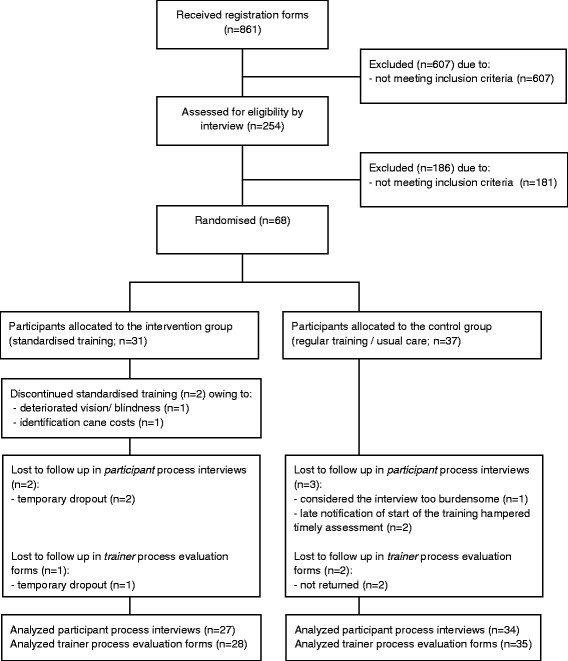
Flow chart of progress of participants and the data collection in the process evaluation.

The flow chart of progress of participants and the data collection in the process evaluation is shown in Figure 1. Of the 861 received registration forms of the local centers, 254 potential participants met the initial criteria. A total of 73 participants that were reported by the local centers fulfilled the final selection criteria. Sixty-eight participants (93%) signed an informed consent form and were officially assigned to the intervention (n = 31) or control group (n = 37). Two participants discontinued the training owing to deteriorated vision (n = 1) and costs related to the purchase of the ID cane (n = 1).

### Self-reported benefit or achievement

#### Trainers

In the process questionnaires, trainers reported on participants’ benefit regarding the training, the use of the ID cane in daily life and the achievement regarding training goals. The overall self-reported benefit regarding the O&M-training was high and according to the trainers the majority of the participants of both trainings perceived benefit with respect to several elements, i.e., experiencing advantages of the ID cane, increased self-confidence, and independent participation outdoors. With regard to achieving the goals ‘optimal use of personal capabilities’ and ‘starting new activities and/or taking up former activities’, the trainers were less convinced that the participants had reached them. Yet, compared with the participants in the regular training group, slightly more participants of the standardised training reached these goals according to the trainers. The trainers expected that 43% and 35% participants of the standardised and regular training group, respectively, would use the cane each day.

### Paticipants

The overall self-reported benefit with respect to the O&M-training according to participants was rather high in both the standardised training group (89%; n = 25) and regular training group (84%; n = 31). The majority, i.e. 79% (n = 22) of the participants in the standardised training group and about half (54%; n = 20) of the participants in the regular training group, reported to use the ID cane in daily life (varying from once a week to daily) during outdoor activities such as crossing a street, crossing an intersection, walking in a crowded area, or travelling by public transport. Additionally, the majority of the participants in the standardised training group reported to use the ID cane on a daily basis, i.e. 89% (versus 54% in the regular training group).

### Experienced barriers and potential solutions according to trainers

In the process questionnaires trainers were asked (i.e. open questions) to report experienced barriers and potential solutions of the training. This part of the questionnaire was completed by 88% (n = 15) of the trainers of the regular training group (i.e. 12% (n = 2) experienced no barriers) and all trainers (n = 12) of the standardised training group.

#### Regular training

Trainers of the regular training group mentioned four important weak aspects of their O&M-training. First, the lack of practicing ID cane use, outdoors (n = 6) or in more complex situations (n = 3). Second, the absence of a telephone follow-up after the last face-to-face session (n = 3). Third, the lack of clear contracting with participant on the format of the training (duration and number of sessions) (n = 3). Lastly, the inability to convince the participant about the importance of using ID canes (n = 3). In contrast, the following positive opportunities regarding the regular training were mentioned: adjusting the training to participants’ needs (tailor-made) (n = 5), raising participant’s awareness of using an ID cane (n = 6) and practicing the use of ID canes (n = 6).

#### Standardised training

Trainers of the standardised training group mentioned one main protocol deviation, i.e. not applying the action plans. According to the trainers, participants understanding of applying action plans hampered the use of it, as well the participant’s or trainer’s belief that action plans were unnecessary. As a result, formulating an action plan as a homework assignment or part of the contracting was also rarely encouraged by the trainers and/or performed by the participants. Furthermore, three main barriers were mentioned by the trainers. First, the standardised training was considered too time and paper consuming (n = 4). Second, the trainers viewed that the standardised format of the training did not meet the different O&M-needs of each participant with regard to the number of sessions (n = 6). Lastly, the applied techniques (e.g., individual problem-solving or prioritizing needs) were considered too demanding for several participants (n = 3). On the other hand, several encouraging factors for the applying the standardised O&M-training were mentioned: the active participation of the participant (n = 4), the applied techniques (i.e. crystallizing and prioritizing participants’ needs, or individual problem-solving) (n = 3), the use and format of a standardised protocol (n = 4), and the content of training, especially the explanation of how to use the ID cane (n = 4).

### Experienced barriers and solutions according to participants

In the process interviews participants were asked (i.e. open questions) to report experienced barriers and potential solutions of the training. This question was completed by 49% (n = 18) participants of the regular training group and 79% (n = 22) participants of the standardised training group. The remaining participants experienced no barriers.

#### Regular training

Three hampering aspects of the regular training as received reported by the participants were the following: the lack of contracting (e.g., agreements on duration and number of sessions) (n = 2), the lack of practicing the use of the ID cane outdoors (n = 1) and the lack of practicing the use of the ID cane in more challenging situations (n = 2). On the other hand, participants mentioned that the training was tailor-made (n = 4) and that the trainer provided adequate information on the use of the ID cane (n = 7). Several participants mentioned that they were able to practice the use of the ID cane (n = 8), that the training improved their feelings of safety, self-confidence and recognition (n = 11), and that the trainer decreased psychological barriers regarding the use of the ID cane (n = 3).

#### Standardised training

Participants mentioned three hampering factors with respect to the received standardised training. First, the standard use of three training sessions (n = 6). Second, the lack of practicing the use of the ID cane in more complex situations, such as a busy intersection (n = 5). And finally, the trainer’s attention for psychological barriers was considered insufficient (n = 3). Nevertheless, three encouraging aspects of the standardised training mentioned by participants were the obtainment of adequate information on the use of the ID cane (n = 7), the ability to practice the use of the ID cane (outdoors) (n = 13), and the increase of feelings of safety, self-confidence and recognition by using of the ID cane (n = 19).

### Acceptability

#### Performance according to protocol

The characteristics of the standardised and regular training, e.g., number of sessions, duration, preparation time, format and location of sessions, are shown in Table 3. The number of sessions differed significantly between both groups (*p* = 0.01). According to the protocol the standardised training mostly consisted of three sessions; the regular training comprised mostly one session. Within both groups a considerable variation in duration of the sessions was reported. In the standardised training group mean duration was evaluated both per session and by a summation of the duration of each session element, which resulted in an inconsistency (partly due to rounding off the time for each element).

**Table 3:** Characteristics of the training per group

	**Standardised training group**		**Regular training group**		***p-value***
**Characteristics of training**	**(n = 28)**		**(n = 37)**		
number of sessions (%)					0.01
1 session	6	(21)	23	(62)	
2 sessions	4	(14)	6	(16)	
3 sessions	17	(61)	6	(16)	
4 sessions	1	(4)	0	(0)	
mean (min-max) duration (in min)*					
session 1	94	(5–135)	70	(10–120)	0.07
session 2	95	(20–100)	60	(5–105)	0.08
session 3	20	(5–90)	54	(15–90)	0.16
Total	209		184		
mean (min-max) preparation time (in min)					
session 1	25	(5–60)	17	(0–60)	0.87
session 2	12	(0–30)	15	(0–30)	0.76
session 3	9	(0–15)	16	(0–30)	0.01
Total	46		47		
format and location of sessions (number (%))					
session 1: face-to-face participants home	25	(89)	33	(89)	
face-to-face low vision center	0	(0)	2	(5)	
session 2: face-to-face participants home	23	(82)	11	(30)	
face-to-face low vision center	0	(0)	2	(5)	
by telephone	0	(0)	2	(5)	
session 3: by telephone	9	(32)	0	(0)	
face-to-face participants home	9	(32)	6	(16)	

Note: all numbers and percentages do not add up to final numbers due to missing data.

* Mean duration reported by trainers of the standardised training group was evaluated per session as well as a summation of the duration of each session element. The latter is shown in this table. The mean duration reported for session 1, 2 and 3 was 78, 85 and 28 minutes, respectively (total mean duration: 191 minutes).

Table 4 presents the duration and extent to which each session element was performed according to the content of the protocol of the standardised training and if the participant actively participated. This data shows that the mean time spent on session 1 and 3 largely corresponds to the time indicated in the protocol, but that on average an extra 15 minutes was spent on session 2. Several deviations from the content of the protocol were reported; deviations mostly concerned the action plans (i.e. formulation, performance and evaluation of action plans) and the contracting.

**Table 4:** Extent to which the standardised orientation and mobility training was performed according to protocol

					**Content protocol**						**Participants**					
**Elements of standardised training**	Mean duration (in min)*				**Performed**		**Partly performed**		**Not performed**		**Active participation**		**Partly participation**		**No participation**	
	**PRT**		**Qt**		**n**	**(%)**	**n**	**(%)**	**n**	**(%)**	**n**	**(%)**	**n**	**(%)**	**n**	**(%)**
Session 1 (n = 28)	90		94													
exploration of participant’s needs		10		19	22	(79)	2	(7)	4	(14)	17	(61)	6	(21)	0	(0)
providing information		10		13	20	(71)	3	(11)	5	(18)	17	(61)	5	(18)	0	(0)
formulating action plan		15		15	15	(54)	5	(18)	7	(25)	10	(36)	6	(21)	3	(11)
performing action plan		30		27	12	(43)	7	(25)	8	(29)	10	(36)	8	(29)	0	(0)
evaluation action plan		10		10	10	(36)	8	(29)	9	(32)	10	(36)	4	(14)	2	(7)
Contracting		15		10	8	(29)	4	(14)	15	(54)	2	(7)	5	(18)	2	(7)
Session 2 (n = 22)	80		95													
general and action plan evaluation		15		18	7	(25)	7	(25)	5	(18)	9	(32)	2	(7)	0	(0)
formulating new action plan		15		9	1	(4)	7	(25)	11	(39)	3	(11)	5	(18)	0	(0)
performing action plan session 1		20		31	4	(14)	4	(14)	11	(39)	3	(11)	4	(14)	0	(0)
performing new action plan		20		25	3	(11)	5	(18)	10	(36)	4	(14)	4	(14)	0	(0)
evaluation and contracting		10		12	9	(32)	4	(14)	3	(11)	8	(29)	2	(7)	0	(0)
Session 3 (n = 18)	25		20													
general evaluation		15		13	9	(32)	7	(25)	1	(4)	13	(46)	2	(7)	0	(0)
Contracting		10		7	7	(25)	1	(4)	7	(25)	7	(25)	0	(0)	0	(0)

Note: all numbers and percentages do not add up to final numbers due to missing data.

* PRT = duration according to the protocol; Qt = duration reported in process questionnaire by O&M-trainer.

The extent to which the participant in the standardised training group achieved the training goals as reported by the trainer is shown in Table 5. Trainers stated that the majority of the participants fully achieved the goals with respect to receiving adequate information on the use of the ID cane, being demonstrated the use of the ID cane, and experiencing the use of the ID cane. Being aware of the advantages of the ID cane was fully achieved by 43% of the participants. According to the trainers the goals regarding phrasing important activities related to mobility, phrasing how to perform these activities safely and independently, and learning orientation and mobility skills were only partly achieved. The goal which was not achieved in nearly 40% of the participants was setting goals regarding action plans.

**Table 5:** Extent to which the participants in the standardised orientation and mobility training group achieved the goals of the training according to the trainer

**Goals of the training**	**Participants (n = 28)**					
	**Achieved**		**Partly achieved**		**Not achieved**	
The participant…	n	(%)	n	(%)	n	(%)
has received information on the use of the ID cane	17	(61)	4	(14)	1	(4)
was aware of the of the advantages of the ID cane	12	(43)	9	(32)	1	(4)
was demonstrated the use of the ID cane	18	(64)	2	(7)	2	(7)
experienced the use of the ID cane	17	(61)	4	(14)	1	(4)
phrased his/her important activities related to mobility	7	(25)	13	(46)	2	(7)
phrased how to perform activities safely and independently	4	(14)	13	(46)	5	(18)
set goals regarding an action plan	1	(4)	10	(36)	11	(39)
learned orientation skills	3	(11)	10	(36)	5	(18)
learned mobility skills	9	(32)	11	(39)	1	(4)

Note: all numbers and percentages do not add up to final numbers due to missing data.

#### Participants’ exposure to and engagement in the training

The majority of participants in the standardised training group received three training sessions (61%), while most participants in the regular training group received one training session (62%) (Table 3). With respect to the use of materials of the standardised training, 83% (n = 10) of the trainers reported the use of the worksheet for prioritizing participants’ needs and the worksheet for action planning (not tabulated). The latter includes techniques such as individual problem-solving and finding personal realistic solutions. About half of the trainers (58%; n = 7) reported the use of the background information on the ID cane, the checklist with the items of the protocol, and the supplementary ID cane. According to the trainers in the standardised training group 7% (n = 2) complied completely with the contracting, 57% (n = 16) complied only partly, and 11% (n = 3) of the participants did not comply at all (not tabulated). Additionally, they stated that 14% (n = 4) of the actions plans formulated by the participants was of high quality and 36% (n = 10) of low quality.

#### Opinion about the training

##### Trainers

Compared with the overall opinion on the O&M-training of the trainers of the regular training group, the trainers of the standardised training group were significantly less positive (Table 6). However, the trainers of the standardised training group gave the participants’ engagement in the standardised training a slightly higher report mark compared with the participants’ engagement in the regular training. About 35% (n = 10) of the trainers of the standardised training group considered the number of sessions (too) much, whilst 35% (n = 13) of the trainers of the regular training group considered the number of session (too) less. Trainers of both groups were generally positive about the duration and the progress of the training and about the extent to which participants’ need for mobility support was met (Table 6). Notably, the trainers rated the participants’ engagement in formulating action plans and carrying them out as part of the standardised training with relatively low average report marks, 4.1 and 5.3 out of a range between 0 and 10, respectively (not tabulated).

**Table 6:** Trainers’ and participants’ opinion about the orientation and mobility training per group

					**Standardised training group (n = 28)**				**Regular training group (n = 37)**				***p-value***^***†***^
**Opinion regarding:**	**Trainer**		**Participant**		**Trainer**		**Participant**			
the overall training*	6.1		8.3		7.5		7.9		0.02
the trainer’s performance*	7.6		8.6		7.5		8.3		0.24
the participants’ engagement in the training*	7.8		7.8		7.4		7.4		0.15
	*n*	*(%)*	*n*	*(%)*	*n*	*(%)*	*n*	*(%)*	
the number of sessions									
(too) much	10	(36)	2	(7)	1	(3)	0	(0)	
Good	12	(43)	24	(86)	20	(54)	30	(81)	
(too) less	3	(11)	1	(4)	13	(35)	0	(0)	
the need for extra sessions	3	(11)	4	(14)	4	(11)	6	(16)	
*the duration of the sessions*									
(too) long	4	(14)	5	(18)	1	(3)	0	(0)	
Good	18	(64)	20	(71)	30	(81)	31	(84)	
(too) short	2	(7)	2	(7)	3	(8)	2	(5)	
*the progress of the training*									
(very) easy	10	(36)	25	(89)	21	(57)	31	(84)	
not easy/not difficult	10	(36)	2	(7)	13	(35)	2	(5)	
(very) difficult	5	(18)	0	(0)	0	(0)	0	(0)	
*the extent to which the participant’s need for mobility support was met*									
not satisfactory	1	(4)	2	(7)	0	(0)	2	(5)	
partly satisfactory	7	(25)	10	(36)	9	(24)	12	(32)	
Satisfactory	17	(61)	14	(50)	25	(68)	20	(54)	

Note: all numbers and percentages do not add up to final numbers due to missing data. * Mean report mark on a scale from 1 (low) to 10 (high). ^†^ Trainers’ opinion of standardised training group compared with trainers’ opinion of regular training group.

##### Participants

Participants of the standardised training group rated the overall training, the trainer’s performance, and their own engagement in the training slightly higher than the participants of the regular training group (Table 6). The vast majority of participants of both groups was positive about the number of sessions and its duration, about 15% indicated the need for an additional session. Progress of the training was reported as rather easy by most (about 86%) participants of both groups. Noteworthy, 36% of the participants who received the standardised training and 32% who received the regular training were only partly satisfied about the extent to which the need for mobility support was met (Table 6). Approximately 90% of the participants in both groups reported that their training was comprehensible and not burdensome, and would recommend the training to other partially-sighted older adults (not tabulated).

## Discussion

This study provides insight into the usefulness (feasibility) and acceptability of a newly developed standardised O&M-training as well as the regular O&M-training, i.e., usual care, in the use of the ID cane as regarded by both partially-sighted older adults and O&M-trainers. Overall, the standardised and regular O&M-training showed similar results and were useful and mostly acceptable for the partiallysighted older adults and O&M-trainers.

Several findings regarding the *usefulness of the training* are noteworthy. To begin with the population reached in the trial (n = 68) was less than expected based on a dossier study by Verstraten [10]. Yet, we included the population that likely corresponds to the visually impaired older adults that will receive this kind of training in the future. Furthermore, the trainers from the regular training group were inconsistent with respect to the experienced barriers and potential solutions that they reported. Although the regular training was defined as tailor-made, it appears that not all trainers optimize the training possibilities, such as practicing the ID cane outdoors or in more complex situations. It is unclear what reasons underlie this lack of optimization by the mobility trainers who essentially have a 'carte blanche' for the training in its entirety, e.g., content, delivery, number of sessions. Additionally, in the standardised training group formulating, performing and evaluating action plans was largely lacking, since only 36 to 54% of the participants were exposed to action planning during the first session and this decreased to 4 to 14% in the second session. Trainers considered the applied techniques, such as action planning, often too demanding for the participant. These downsides of the intervention may be caused by two factors. First, trainers may have been insufficiently familiar with behavioural change techniques in general and using action plans in particular due to the limited time for instruction on the standardised training and the limited number of participants per trainer. Second, trainers may not have been convinced of the need to use action plans since they had not experienced its benefits for participants. Further, trainers considered the standardised training too time and/or paper consuming. This may also be explained by the limited time for instruction of the mobility trainers and the limited application of the standardised training by the mobility trainers, as during the instruction it was stressed that the written protocol and its forms should be considered as a guidance until the trainer gained sufficient experience to know the training method by heart. Lastly, although participants considered the trainer’s attention for psychological barriers insufficient, they reported an increase of feelings of safety, self-confidence and recognition by using the ID cane. These positive effects of the trainings reported by participants are in accordance with other studies evaluating low vision rehabilitation in general [20-22]. For instance, Engel et al. demonstrated an increase in self-confidence of partially-sighted older adults in their daily life activities after an O&M-training program [21]. Eklund and colleagues found strong evidence for an ADL health education programme for partially-sighted older adults to enhance security and to hinder a progressive decline in perceived security in ADL [22].

Regarding the *acceptability of the training* the extent to which the intervention was performed according to protocol, the participants’ exposure to and engagement in the training, and the opinion about the training were assessed. Nearly 40% of the participants of the standardised training group were not exposed to the training according to protocol with respect to the number of sessions, the telephone follow-up, formulating, performing and evaluating action plans, or contracting. These deviations from the protocol may be reflected in the low quality of the formulated actions and the lack of compliance regarding contracting by the participants. The goals of the standardised training were mostly either partly or not at all achieved. This is reflected in the almost 40% of the participants who did not manage to set goals regarding action plans according to the trainers. Trainers were fairly positive about the active engagement of the participants in the training though. Trainers’ overall opinion about the standardised training was inconsistent among trainers, but significantly lower than the overall opinion of trainers in the regular training group. Participants of both conditions were, however, positive about the training and the trainer. It is worth mentioning that only about half to two-thirds of the trainers and the participants of both training groups was satisfied with the extent to which the participant’s need for mobility support was met (Table 6). This indicates that there is still a large gap between the participants’ needs regarding mobility and the extent to which the O&M-training satisfactorily meet these needs.

Along insight into the usefulness and acceptability of the O&M-training, this process evaluation may help to explain the observed effects, to recognize strong and weak aspects of the intervention, and to implement the standardised O&M-training, if it turns out to be effective [16,17]. To explain the observed effects it should be reconsidered to what extent the standardised training differed from the regular training. With respect to characteristics of both conditions we found no significant difference in preparation time or duration of the training, only the number of sessions significantly differed. Compared with the regular training the standardised training had several advantages, such as an active role of the participant, the use of a structured protocol which provides an equal starting point for all trainers, and the inclusion of follow-up care, which were all acknowledged by the trainers. Additionally, the standardised training provided a clearly stated general goal of the training, which was generally lacking in previous descriptions of this kind of training [11] and was aimed at self-management and behavioural change techniques, such as action planning, problem-solving and contracting which have previously shown successful in other populations [23-25]. However, our data suggests that the latter distinguishing elements were scarcely applied by the trainers of the standardised training. Therefore, if no differences between both groups, e.g., regarding mobility and activities of daily life, are observed in future effect analyses this lack of performance according to protocol may be regarded as a potential cause.

Hence, a weak aspect of the intervention is the difficulty trainers experienced in conducting self-management and behavioural change techniques during the training. Trainers who facilitated the standardised training received a two-hour instruction on the use of the protocol, which was self-explanatory. However, this instruction was most likely still insufficient for the following reasons: trainers were not used to work according to a protocol and trainers were not familiar with the techniques. Additionally, researchers did not monitor the training sessions to observe if trainers followed the protocol and there was no intermediate feedback of trainers to the researchers about the lack of using action plans, contracting, or other techniques. The aforementioned weak aspects are points of interest for future studies. Given the rather difficult inclusion of participants, during a much longer than expected period of time, the study population of the present study was relatively small and trainers facilitated the training for only one to four participants. This may have hampered the performance of the training according to protocol because knowledge and skills, e.g., regarding self-management and behavioural change techniques, as obtained during the instruction may have been lost given 1) the substantial time between instruction and actual application of the training and 2) insufficient exposure to the standardised training. In view of most trainer’s ample experience in conducting O&M-training, we assume that the trainers reverted to their usual training style and content. This is reflected in the data, which indicates, for example, that the intervention parts for which the standardised training differs from the regular training the trainers only partly achieved their training goals.

The poor opinion about action planning as observed in this study is inconsistent with other studies [26,27]. For instance, Smeulders et al. and Rees et al. found that the use of action planning was considered a strong point of their cognitive-behavioural self-management group intervention according to both participants and facilitators [26,27]. In addition to the limited instruction and lack of exposure of the trainers to action planning in our study, this inconsistency may be explained by the formats of the interventions as applied in both studies. These were group-based, which not only provides an opportunity for social interaction, but also enables participants to share experiences and coping strategies [27,28]. The supportive telephone calls from co-participants to motivate each other in formulating and performing action plans in the study by Smeulders et al. may have contributed to an appreciation of the use of action plans as well [27].

The potential implementation of the standardised O&M-training in the use of the ID cane in the Netherlands will be performed in close consultation with the Dutch Low Vision Health Care centers Bartiméus and Royal Dutch Visio. Previous consultations with these centers have resulted in an implementation and dissemination plan which mainly consists of embedding the standardised training in the Dutch national instruction for O&M-trainers organized by both centers collectively. Implementation costs can be estimated by the time and labor needed by the centers. If the standardised training protocol is implemented in the Dutch National Low Vision Health Care, we recommend providing an extensive train-the-trainer program with follow-up contacts for O&M-trainers and improving the protocol based on the experiences in the current study. Trainers should be able to incorporate self-management and cognitive behavioural techniques into the training (e.g., action plans, problem-solving, cognitive restructuring) and should stimulate participants to perform homework activities (contracting).

## Conclusions

Overall, this process evaluation provides insight into the usefulness (feasibility) and acceptability of the standardised O&M-training as well as the regular O&M-training, i.e., usual care according to partially-sighted older adults and O&M-trainers. We may conclude that both training conditions showed to be useful and mostly acceptable for the partially-sighted older adults and trainers. Partially-sighted older people appreciated both trainings and perceived benefit. Regarding the latter the standardised training showed slightly better results. Yet, a major concern is the observed deviation from the protocol of the standardised O&M-training by the O&M-trainers regarding several elements of this training that should distinguish the standardised training from the regular training. This may hamper potential effects on mobility, ADL self care and other outcomes studied in the effect evaluation. Future O&M-training should to a larger extent meet the mobility needs of the partially sighted older adults. Further instruction and follow-up of the performance of O&M-training of the mobility trainers may contribute to improved care within the Dutch centers for low vision.

## Competing interests

The authors declare that they have no competing interests.

## Author’s contributions

GZ, GvR, JS and GK contributed to the conception and design of the study. JB and GZ drafted the manuscript with input from the other authors. All authors read and approved the final manuscript.

## Pre-publication history

The pre-publication history for this paper can be accessed here:

http://www.biomedcentral.com/1472-6963/12/141/prepub
